# Attenuation of Rat Colon Carcinogenesis by *Styela plicata *Aqueous Extract. Modulation of *NF-κB* Pathway and Cytoplasmic *Sod1* Gene Expression

**DOI:** 10.31557/APJCP.2020.21.9.2739

**Published:** 2020-09

**Authors:** Elsayed I Salim, Mona M El-Gamal, Mahy M Mona, Hanaa A Abdelhady

**Affiliations:** 1 *Genetics and Cancer Research. Research Lab. of Molecular Carcinogenesis, Zoology Department, Faculty of Science, Tanta University, Tanta, Egypt. *; 2 *Zoology Department, Faculty of Science, Tanta University, Tanta 31527- Egypt. *

**Keywords:** Styela plicata, colon cancer, aberrant crypt foci, NF-kappa B, Cu-Zn superoxide dismutase

## Abstract

**Objective:**

In search for a unique natural combination of highly active biological components for treatment against colon cancer, we used aqueous extract of Ascidia, Styela plicata (ASCex), a marine invertebrate depending on its richness of high levels of biologically active components as indicated in our previous studies, against rat colon cancer, exploring its underlying mechanisms.

**Methods:**

Rats chemically initiated for colon cancer were either non-treated or post-treated with highly saturated ASCex for 32 weeks after initiation, other groups of rats were administered ASCex without cancer initiation or served as normal controls.

**Results:**

Rats treated with ASCex alone did not show any signs of non-favored health conditions. Treatment with ASCex after cancer initiation has significantly reduced the average incidences, multiplicities and volumes of colon tumors (adenomas and adenocarcinomas) as compared with the non-treated cancer group. ASCex has also significantly reduced the total numbers of aberrant crypt foci (ACF), surrogate biomarkers for colon cancer as compared with the non-treated cancer group. Moreover, anti-proliferative celluar nucular antigen (PCNA) immunohistochemical staining revealed that ASCex exerted significant antiproliferative characteristics in the carcinogen-treated colonic mucosa as compared with its corresponding control. Also, treatment with ASCex has markedly down-regulated the mRNA expression levels of Nuclear Factor-kappa B (NF-κB), a nuclear transcriptional activator as well as the mRNA expression of the cytoplasmic SOD1 gene which encodes Cu/Zn SOD, the first line defense against superoxide radicals.

**Conclusion:**

Collectively, ASCex could act as a potent chemotherapeutic drug against colon cancer, likely through the influence of its rich active metabolites which interfere with various biological pathways including inhibition of protein synthesis during cellular growth and marked induction of antioxidative capacity in the colonic mucosa. This role has been extensively discussed herein.

## Introduction

Marine creatures denote source of bioactive secondary metabolites molecules, that could be prospective candidates for various disease medications (Senthilkumar and Kim, 2013), these include potent antioxidants (Singh et al., 2016), antimicrobial (Maddox et al., 2010), antiviral (Dang et al., 2015), antibacterial (Meenakshi, 2012), antifungal (Plaza et al., 2009), antiparasitic (Wei et al., 2010) and antitomour resources (Sawadogo et al., 2013). The national cancer institute (NCI) of the USA evaluations assume that about 1% of marine natural products (MNPs) exhibiting anti-tomour or cytotoxicic properties as against only 0.01% amongst their terrestrial equivalents (Satheesh et al., 2017). Previously, a significant well known soft tissue sarcoma natural remedy, Yondelis®, was isolated from Acteinascidin (type of ascidia). Also Aplidin® isolated from Aplidium albicans demonstrates ability in decreasing tumours of different types of cancers (Adrio et al., 2007), while other MNPs are still undergoing clinical trials such as plitidepsin and lurbinectedin (Meyer, 2017).

Ascidians or sea squirts are known as tunicates due to the polysaccharide containing tunic that envelops the animal and forms a somewhat flexible skeleton, found tied to rocks and high current turfs. Nearly, 3,000 living species of ascidians were reported (Shenkar and Swalla, 2011), representing the highest cluster of marine species frequently investigated for natural products and deliver opulent value of bioactive secondary metabolites with compelling biomedical uses (Lawal et al., 2016).

Colorectal cancer (CRC) is one of the most frequently diagnosed malignant diseases, and one of the leading causes of cancer related deaths, occupies rank 10th among most common cancers in the world in males and 12^th^ in females of all ages, with age standardized incidence and mortalities of 20.6/100.000 and 10/100.000 in males and females respectively (Ferlay et al., 2013). In Egypt, this disease has been increasing in the recent decades (Abd ElLateef et al., 2020) due to many etiological factors such as high fat diets, high red meat consumption, less physical activities and increased diabetes among younger populations, where it occupies rank 8 among most common cancers in both males and females, with age standardized incidences and mortalities of 6.1/100.000 and 4.1/100.000 in males, and 5.2/100.000 and 3.4/100.000 in females respectively (Ferlay et al., 2013).

The reactive oxygen species (ROS) radicals are generally eliminated by antioxidative enzymes. Mammalian cells are equipped with both enzymatic and non-enzymatic antioxidant mechanisms to minimize the cellular damage that results from interactions between cellular constituents and ROS. Superoxide dismutases (SODs) constitute the first line of defense against superoxide radicals. SODs that catalyze the conversion of reactive superoxide anions into H_2_O_2_, which is an essential ROS (Kim et al., 2015) are three known groups, according to the metal co-factor within the enzyme; Fe SOD, Mn SOD and Cu/Zn SOD. These three groups are encoded by different genes. The gene Sod1 encodes the cytoplasmic Cu/Zn SOD enzyme (Tower, 2015). Cu/Zn SOD functions as a homodimer and is found both in the cytoplasm and the outer mitochondrial space (Deepa et al., 2019). The gene Sod2 encodes the Mn/SOD enzyme. Mn/SOD functions as a tetramer and is restricted to the innermost mitochondrial space (Tower, 2015). The gene Sod3 encodes the extracellular form of Cu/Zn SOD, sometimes called ecSOD (Oberley-Deegan, et al. 2009).

Nuclear factor kappa- B (NF-κB) is a protein responsible for DNA transcription and, cytokine manufacturing and cell integrity. Most animal cells contain NF-κB which elaborates cellular defense against various stimuli such as free radicals, toxic heavy metals, UV light and others (Perkins, 2007). It is important in controlling cellular reactions as it is a rapid-acting primary transcription factor i.e., transcription factors that are present in cells in an inactive state and do not need new protein synthesis in order to become activated (other members of this family include transcription factors such as c-Jun, STATs, and nuclear hormone receptors). This allows NF-κB to be a first defense barrier for damaging cellular inducements mainly ROS (Liu et al., 2020).

Recently, we reported several metabolites in the aqueous and ethanolic extracts of the Egyptian Styela plicata obtained from the Mediterranean coast (EL-Gamal et al., 2018). In this context, the water extract confirmed the presence of 25 chemical constituents using HPLC analysis of which the majorities were Dermatansulphate, Kuanoniamine A, lissoclinamide, palmitic acid, stearic acid, Paromomycin and glycine sulphate. While the ethanolic extract confirmed the existence of another main chemical constituents using gas chromatography/Mass chromatography (GC-MS2) analysis of which the majorities were Actinobolin, Palmitic acid and Floxurdin. In addition, the sub-acute toxicity test (28-days) for graded concentrations of the aqueous extract on male Sprague–Dawley (SD) rats (EL-Gamal et al., 2018) showed no toxic or pathologic symptoms, so that was a preface for the present experiment about testing the extract as a candidate therapy for colon cancer in rats and to estimate it’s underlying molecular mechanisms.

## Materials and Methods

Healthy male 6 -week-old Sprague-Dawley (SD) rats, acquired from The Holding Company for Biological Products & Vaccines (Vaccera), Giza- Egypt, were assigned to plastic cages with saw dust for bedding, covered with metal networks and indorsed to adapt for one week in the animal facility environments at the Faculty of Science, Tanta University, before experimentation. All experimental protocols were in accordance with the Guidelines for Ethical Care of Experimental Animals. The institutional animal care and use committee (IACUC) and ethical committee of the Faculty of Science, Tanta University, Egypt permitted the experiment under the approval number of IACUC-SCI-TU-0083. Levels for temperature and humidity were 23±3°C and 52±5% respectively, with natural day/night cycle. The animals were offered drinking tap water and well-adjusted animal food ad libitum. Body weights, food consumption and water intakes were precisely recorded every week.

*Initiation of rat multi-step colon carcinogenesis*

1,2-Dimethylhydrazine (DMH) was purchased from Sigma-Aldrich, St. Louis, MO, (USA). At the beginning, animals were given 8 consecutives subcutaneous (S.C.) injections of DMH, once a week, dissolved in physiological saline solution at a dose of 40 mg/kg body weight. Rats were treated with ASCex and observed for 32 weeks after the last DMH injection.

*Preparation of S. plicata extract*

S. plicata aqueous extract was prepared as previously described (El-Gamal et al., 2018). Briefly, Styela plicata samples collected from Alexandria port, North of Egypt were recognized according to Monniot et al., (1991). Adherents were detached and specimens were washed with seawater, cut into small parts, dried, and homogenized then dried to get a coarse powder, stored in an air fitted bottle. Fresh doses were prepared at use by 9 grams of Ascidia powder dissolved in 45 ml of distilled water heated in water bath at 45°C, stir, let overnight in dark area, filtered then used.

*Experimental protocol*

**Figure 1:**
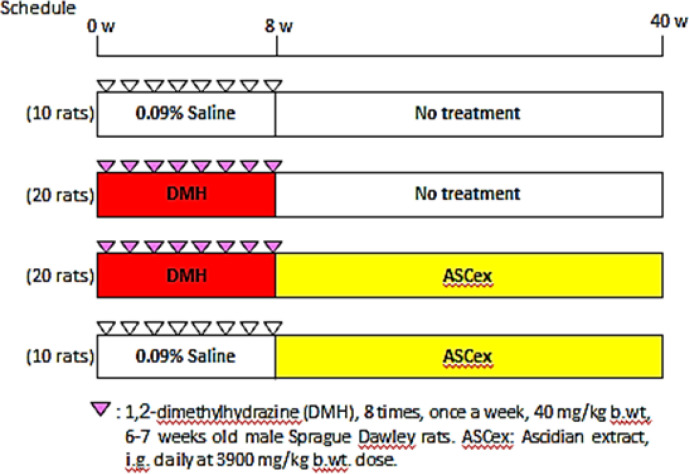
Experimental Protocol

Rats were divided into four groups as follows: Group 1 (10 rats) normal control injected with the vehicle (0.09% saline). Group 2 (20 rats) were injected with DMH as described above and served as positive cancer control. Group 3 (20 rats) were injected with DMH then daily (6 days per week) intragastroluminally (i.g.) given with fresh S. plicata water extract (ASCex) at a calculated dose of 3900 mg/kg body weight (1ml/day)20. Group 4 (10 rats) were i.g. administered with the same dose of ASCex as described in group 3 but without DMH administration (experimental protocol, Figure 1).

All rats were sacrificed under surplus of ethyl ether anesthesia after 40 weeks from start, blood was collected from the abdominal aorta of all sacrificed rats in 10 ml normal tubes, centrifuged at 3,000x, sera were gathered and frozen at -20^o^C until use. Pathological examinations were achieved macroscopically on all rats during sacrifice. Absolute and relative organ weights (organ wt./b.wt. x 100 were taken for vital organs such as liver, kidneys, spleen and testes. Specimens from these organs as well as from colons were preserved in 10% phosphate-buffered formalin. Anomalous masses were collected from the colons and parts of it were kept in the same fixative until routinely prepared for histopathology while the other parts were preserved in -86^o^C for the biochemical and molecular analyses. Tumor volumes (v), were estimated with calipers and considered by to the equation: V= (L X W2/2) (Shen et al., 2003). The lesions were pathologically diagnosed by two animal pathologists according to Turosov and Mohr, (1990).

*Preparation of the colon and ACF counting*

At sacrifice, the whole colon was excised, inflated then cut along longitudinal access and rinsed in saline. For ACF assay, the proximal, middle or distal colonic segments were cut and fixed flat in 10 % phosphate buffered formalin, rinsed then stained with 0.2% methylene blue for 1-2 min for ACF visualization by two investigators under a light microscope. 5 whole colons from group 1, 13 from group 2, 14 from group 3 and 5 from group 4 were used for the ACF assay, while the remaining colons in each group were assayed for the molecular and biochemical analyses tests (see below). Foci with one aberrant crypt (1AC), 2ACs, 3ACs, or larger ones ≥4ACs were counted and categorized. After ACF determination, small parts of the colonic segments were prepared for histopathology or immunohistochemistry.

*PCNA immunohistochemistry*

4-5µm paraffin sections were prepared, de-waxed, rehydrated then microwaved in sodium citrate buffer. The slides were then cooled and washed in TBS. Slides were then incubated in 3% H2O2 in methanol to minimize endogenous peroxidise action and then subjected to Ultra block (Ultra Vision plus Detection System, Thermo Scientific, USA) to stop non-specific bindings. After washing in TBS, slides were kept overnight at 4^O^C with the PCNA Rabbit polycloned antibody (Product. No. SAB2108448, Sigma Aldrich) at dilution of 1:1000 in a moistened box, then rinsed in TBS. Afterwards, sections were raised with the secondary antibody (Biotinylated goat anti-polyvalent Ultra Vision plus Detection System, Thermo Scientific) for 1 hr then washed in TBS. The sections were again incubated with streptavidin peroxiddase plus then were washed in TBS and developed with 3, 3’-Diaminobenzidine (DAB) solution. Counterstaining was committed with hematoxylin.

*Gene expression analysis using Quantitative Real-Time PCR (qRT-PCR)*

Colon mucosal cells were scraped with autoclaved glass slides from five animals from each group at sacrifice, snap-frozen in liquid nitrogen then kept frozen in -86^O^C for subsequent investigations. Total RNA was extracted using a total RNA isolation kit (analytikjena-Germany) according to the manufacturer’s protocol. The primers’ sequences for the Cu-Zn SOD , NF-κB genes and GAPDH housekeeping gene are shown in Table 1. All the reaction tubes were loaded into a thermal cycler (Applied Biosystems, USA). All primer pairs were synthesized by Jena Bioscience GmbH (Jena, Germany). CU-Zn SOD, NF-κB and GAPDH rat primer sequences for qRT-PCR were brought from the National Center for Biotechnology Information (NCBI) Gene bank (Pubmed) and aligned against each of gene sequences to check their potential amplification products. The concentration of the extracted RNA was spectrophotometrically determined by measuring the absorbance of the solution at 260 nm (RT Step1 nanodrop, Applied BioSystems. USA).

Single stranded complementary DNA (cDNA) was obtained from 1 μg of purified RNA using the Sensiscript Reverse Transcriptase (QIAGEN, Germany) Synthesis Kit, according to the manufacturer’s instructions. qRT-PCR analysis was performed on Rotor gene 5plex, Qiagen, Germany. Analysis was performed with Light Cycler 480 SYBR Green I Master (Roche, Welwyn Garden, Swiss).

The Threshold cycle (CT cycle) was used to determine the expression level in control samples and those treated with ASCex. The gene expression level was calculated as described by Yuan et al., (2016) using Applied Biosystems Step One™ Instrument software. The results were expressed as the ratio of reference gene to target gene by using the following formula: ΔCT (cycle numbers at the threshold level of log-based fluorescence normalized to endogenous control genes) = CT (target genes) - CT (endogenous control genes). To determine the relative expression levels, the following formula was used: ΔΔCT = ΔCT (Treated) - ΔCT (Control). Thus, the expression levels were expressed as n-fold differences relative to the calibrator (RQ; Real-time quantitative PCR). The value was used to plot the expression of the dedicated genes using the expression of 2-ΔΔCt. All samples were repeated in duplicates for data confirmation.

*Statistical analyses*

Groups data expressed as means ± S.D. were analysed using the two-tailed t-test or ANOVA analyses using SSPS software, ver. 17, USA.

## Results

**Figure 2:**
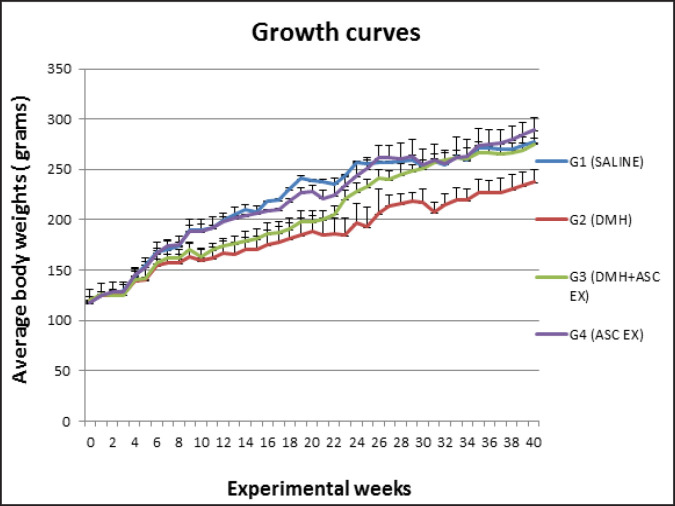
Average Body Weight Curves for Rats in Different Experimental Groups. Ascex, Ascidia Extract

Average body, absolute and relative organs weights Growth curves for rats in all groups are shown in Figure 2. Two rats died from group 2 at weeks 36 and 37 and one rat from group 1 at week 32 for unknown reasons. The average final body weights, absolute liver and kidneys weights as well as calculations for their relative organ weights (%) for rats in all groups are shown in Table 2. No significant body weight differences were observed in all rats except that of rats administered DMH only in group 2 which was lower than the normal control in group 1. Relative and absolute organ weights remained constant among the group.

*Blood analysis*

The haematological parameters of complete blood count (CBC) are shown in Table 3. Administration of DMH alone in group 2 has significantly decreased the RBCs, platelets, Lymphocytes’ counts as compared to normal controls. Treatment with ASCex did not recover total numbers of RBCs after DMH, but recovered platelets counts to almost normal. Other CBC parameters were not changed among the groups. Treatment with ASCex alone in group 3 did not cause any unfavorable conditions to the animals’ blood counts.

The serum biochemical parameters for liver and kidney functions as well as for lipid profile are shown in Table 4. DMH administration in group 2 has increased the Aspartate aminotransferase (AST) levels and decreased the total protein and albumin levels as compared with normal, however, treatment with ASCex after DMH in group 3 declined these levels to almost intact values. Urea levels are observed declined in groups 3 and 4 as compared with controls while creatinine values are not changed. For lipid profile, treatment with ASCex after DMH in group 3 has reduced the DMH-induced high-density lipoprotein (HDL) high levels to normal and sharply reduced the cholesterol and low density lipoprotein (LDL) blood levels. The same effect is noticed after treatment with ASCex without DMH in group 4.

*Effect of ASCex on average numbers of ACF*

All assayed animals that received DMH have shown variant numbers of ACF with different crypt multiplicities distributed in all parts of the colons. No ACF were identified in the colons of normal control rats of group 1 (Table 5). The average total numbers of ACF in group 2 administered DMH only was 124±34.3 ACF per rat. Treatment with ASCex in group 3 has sharply reduced the total ACF numbers by 78.1% into 27.2 ACF per rat. Different criteria of ACF (crypt multiplicities) were also found significantly reduced by ASCex treatment; by 55.9% in 1ACs, while the other criteria of ACs particularly larger crypts with 2ACs, 3ACs, ≥4ACs were surprisingly inhibited by 100%.

*Effect of ASCex on tumors’ incidences and multiplicities*

**Figure 3:**
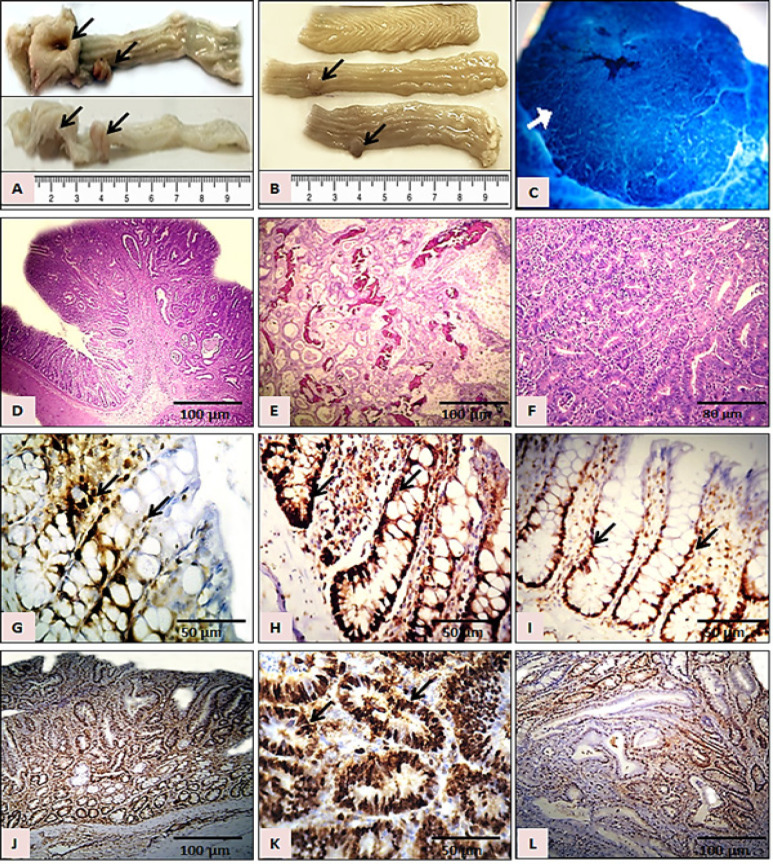
Photomicrographs of: A, Whole colons with large colorectal tumours from group 2 (10X); B, Whole colons with relatively smaller colonic tumours from group 3 treated with ASCex (10X); C, A small microscopic tumour seen in distal colon (40X).(Methylene blue); D, Hyperplastic polyp (H&E); E, Signet- ring cell mucinous carcinoma.(H&E); F, Invasive adenocarcinoma (H&E); G, Normal colonic mucosa (group 1) with moderate numbers of PCNA-labeled cells (arrows).(PCNA); H, Colonic mucosa from group 2 with high numbers of PCNA- positive cells (arrows).(PCNA); I, Colonic mucosa from a rat treated with ASCex only showing ordinary numbers of PCNA- labeled cells (arrows).(PCNA); J, Dysplastic polyp from group 2 showing high numbers of PCNA- positive cells.(PCNA); K, Invasive adenocarcinoma from group 2 showing high numbers of PCNA- positive cells (arrows).(PCNA); L, Invasive adenocarcinoma from a rat treated with ASCex after DMH showing relatively lower numbers of PCNA- positive cells.(PCNA)

Histologically, various types of polyps and tomours were formed in the colons of DMH-administered rats, those were mainly diagnosed as Hyperplastic polyps, adenomatous polyps, dysplastic polyps, tubular adenoma, adenocarcinomas, invasive adenocarcinoma, Signet ring cell carcinoma, mucinous carcinoma, enlarged lymphocytes and carcinoma in situ (Figure 3, A-F).

*Tomours’ incidences (%), multiplicities and volumes*

The tumor incidences were significantly reduced from 93.8% in-group 2 administered with DMH only to 26.7% in rats treated with ASCex after DMH in-group 3 (Table 6). Also, a significant reduction in the tomour multiplicities (mean numbers of tomours per all rats in test groups) was obtained. Importantly, the tomour volumes were also significantly reduced after ASCex treatment in rats of group 3 as compared with the volumes of tomours obtained from group 2.

*PCNA-LI (%) in colonic epithelium*

**Figure 4:**
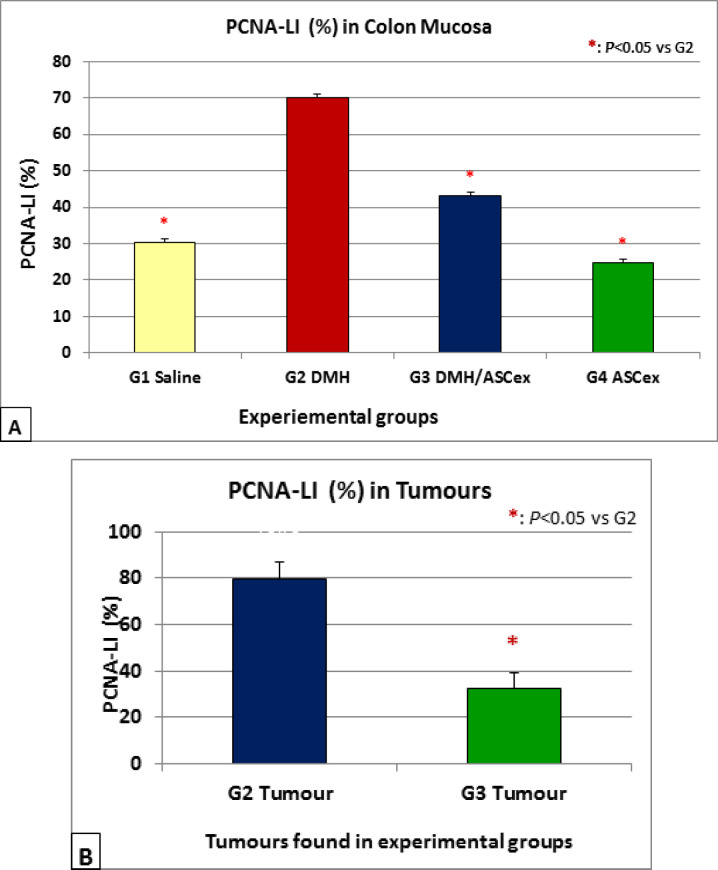
Average PCNA-LI (%) in A, Normally appearing mucosa for all groups; B, Tumours from groups 2 (administerwed with DMH only comparing to tumours from G3 treated with ASCex after DMH administarations. Average PCNA-LI (%) values are calculated in adenocarcinomas only. *: Significant vs. G2 at P<0.05

PCNA immunohistochemical staining showed reddish brown nuclei mainly localized in the lower one third of each colonic crypt of the normal control animals, or extended towards the upper parts of the DMH-administered colonic crypts, while was distributed in all regions of the PCNA-stained tumors (Figure 3, G-L). The average PCNA-LI (%) of the colonic regions of all experimental groups are represented in Figure 4 (A and B). In general, the PCNA-LI (%) were markedly higher in all DMH-administered normally-appearing colonic mucosa over those from control animals (P<0.05). Treatment with ASCex after DMH administration in group 3 has significantly decreased the PCNA-LI (%) in colonic epithelium. However, the group treated with the ASCex only (Group 3) showed almost similar numbers of PCNA-stained nuclei as those seen in the saline control group 1. Average PCNA-LI (%) counts in adenocarcinomas collected from group 3 treated with ASCex after DMH was also significantly inhibited as compared with that of group 2 administered DMH only (Figure 4, B).

*Cu-Zn SOD and NF-κB gene expression data in colonic epithelium*

Results for CU/Zn SOD and NF-κB genes mRNA expression levels in the colonic epithelium of rats treated or not with DMH and/or ASCex after 40 weeks assessed by qRT-PCR analysis relative to GAPDH internal control gene are summarized in Tables 7 and 8.

*Superoxide dismutase (SOD1) gene (Cu/Zn SOD) expression*

Table 7 represents differences in the *Cu/Zn SOD* gene activity levels after the effects of DMH and/or ASCex on rats as compared with their corresponding controls. Treatment with DMH in group 2 showed a significant increase in the Cu/Zn SOD activity as compared with the data of normal control (P<0.05). In contrast, a significant decrease in the Cu/Zn SOD activity was observed in the mucosa treated with DMH+ ASCex after DMH administration in group 3 as compared with that of group 2 (P= 0.045554). Also, ASCex treatment without DMH (group 4) has caused a significant increase in the Cu/Zn SOD activity as compared with the normal control levels (P = 0.046578).

*NF-κB gene expression*

The normalized *mRNA* expression of NF-κB was found significantly elevated in group 2 after administered with DMH only after 40 weeks by 1671131.25-fold as compared with the normalized normal control *mRNA* expression. ASCex treatment after DMH has significantly reduced the *mRNA* expression in the colonic epithelium by 47.21% (956036.955). However, treatment with ASCex alone in group 4 showed mRNA expression levels almost similar to the expression in group 3 (916359.675) (Table 8).

**Table 1:** Primers’ Sequences

Gene	Rat primer sequence (5'–3')	PCR product (bp)	Gene Bank Accession Number
*GAPDH *	F: 5’-TGCAACTAGGATGGTGTGGCT-3’	630 bp	NM_017008
	R: 5’-TCCCATTCCCCAGCTCTCATA-3’		
*CU/ZnSOD*	F: 5′-GCAGAAGGCAAGCGGTGAAC-3′	447 bp	NM_017050
	R: 5′-TAGCAGGACAGCAGATGAGT-3′		
*NF-κB*	F: 5’-CCTAGCTTTCTCTGAACTGCAAA-3’ R: 5’-GGGTCAGAGGCCAATAGAGA-3’	528 bp	AB_302977

F, Forward ; R, Reverse

**Table 2:** Initial and Final Average Body Weights, Absolute and Relative Organ Weights

Group/treatment	G1 / Saline	G2 / DMH	G3 / DMH→ASCex	G4 / ASCex
Initial No. of rats	10	20	20	10
Final No. of rats	10	18	19	10
Initial b.wt. (g)	118.6±5.61	119.9±11.52	120.7±3.14	118±6.27
Final b.wt. (g)	277.5±3.40^a^	237.7±12.06*	275.4±13.12	289.3±12.47
Liver wt. (g)	7.60±1.27 (2.74)^b^	7.16±2.27** (3.01)	8.08±3.58 (2.93)	8.26±0.01 (2.85)
Spleen wt. (g)	0.68±0.05 (0.24)	0.67±0.21 (0.28)	0.75±0.35 (0.27)	0.70±0.07 (0.24)
R.kidney wt. (g)	0.94±0.09 (0.34)	0.76±0.26*,** (0.32)	0.83±0.42 (0.3)	0.86±0.03 (0.3)
L. kidney wt. (g)	0.87±0.08 (0.31)	0.72±0.27*,** (0.3)	0.81±0.38 (0.29)	0.81±0.01 (0.28)
Testes wts. (g)	3.28±0.21 (1.18)	2.84±1.22 (1.2)	3.09±1.66 (1.12)	3.06±0.03 (1.06)

^a^, Values of means ± S.D. are absolute numbers; ^b^, Values between parenthesis are relative organ wts.= Ratio of organ wt./body wt. X100 (%); *, Significant vs. G1 at P<0.05; **, Significant vs. G2 at P<0.05. ASCex: Ascidia extract.

**Table 3:** Complete Blood Count (CBC) for Rats in All Experimental Groups after 40 Weeks

Group	G1 / Saline	G2 / DMH	G3 / DMH+ASCex	G4 / ASCex	Unit
n	10	10	10	10	Rats
WBCs	4.91±1.57	6.89±2.98	5.97±1.13	5.52±1.92	X10³ /μL
RBCs	8.32±0.95	6.85±0.52*	6.65±0.90*	8.52±2.33	X106 /μL
HGB	13.46±2.15	14.74±1.33	13.98±2.08	13.09±4.39	g/dl
HCT	50.58±5.59	48.74±5.11	45.65±8.96	43.96±14.96	%
PLT	1655±279.0	985±193*	1364±349**	1222±447**	X10³ /μL
NEUT	16.60±2.07	20.57±3.64	20±3.79	17.7± 6.19	X10³ /μL
LYM	79.00±2.83	72.14±4.91*	72.50±6.35	69.99±22.64	X10³ /μL
Mon	3.00±0.71	4.71±1.80	5.17±4.83	4.53±3.08	X10³ /μL
Eos	1.40±0.55	2.43±0.79	2.33±1.97	2.25±1.60	X10³ /μL

*, Significant vs. G1 at P<0.05; **, Significant vs. G2 at P<0.05; WBCs: White blood cells; RBCs, Red blood cells; HGB, Hemoglobin; HCT, Hematocrit; PLT, Platelets; NEUT, Neutrophils LYM, Lymphocytes; Mon, Monocytes; Eos, Eosinophils; *n*, No. of test samples.

**Table 4:** Liver, Kidney Functions and Lipid Profile Parameters after 40 Weeks

Group/ treatment	G1 / Saline	G2 / DMH	G3 / DMH+ASCex	G4 / ASCex	Unit
Liver function parameters:					
ALT	93.20±9.23	123.14±5.57*	102.33±23.58	97.87±34.80	IU/I
AST	142.60±5.41	146.57±63.11	131.50±70.38	117.36±48.29	IU/I
ALK	327.80±20.99	317.71±21.69	306.17±15.52	273.89±93.68	IU/I
T. bilirubin	0.71±0.04	0.65±0.06	2.40±4.10	5.51±17.02	mg/dl
D. bilirubin	0.13±0.01	0.12±0.02	0.14±0.04	0.12±0.05	g/dl
T. protein	11.06±1.04	8.47±.83*	8.94±1.21	7.92±2.68	g/dl
Albumin	5.00±0.16	3.67±0.42*	4.50±0.73	3.72±1.29	g/dl
Kidney function parameters:					
Urea	50.80±5.40	53.14±8.47	41.50±8.89**	41.87±14.74	Mg/dl
Creatinine	0.79±0.03	0.71±0.18	0.72±0.18	0.60±.23	Mg/dl
Lipid profile parameters:					
TG	37.80±4.87	35.43±7.66	50.33±29.60	41.06±21.77	Mg/dl
CHO	68.20±3.70	61.43±12.49	50.17±5.56**	51.37±17.26	Mg/dl
HDL	19.20±1.92	15.86±2.97*	13.83±3.49	13.45±4.69	Mg/dl
LDL	42.44±3.22	38.40±13.13	26.27±5.51**	30.47±10.75	Mg/dl

*, Significant vs. G1 at P<0.05; **, Significant vs. G2 at P<0.05; Numbers of samples investigated = 10. ALT, Alanine amino transaminase; AST, Aspartate aminotransferase; ALK, Alakaline phosphatase.TG, Triglyceride; CHO, Cholesterol; HDl, High density lipoprotein; LDL, Low density lipoprotein; No. of test samples is 10.

**Table 5:** Average Total Numbers of ACF in the Colons of Rats Treated or Not with DMH, and/or ASCex after 40 Weeks

Group	Treatment	n	Total No.	No. of Aberrant Crypts per Focus			
			ACFa	1AC	2ACs	3ACs	≥4 ACs
G1	Control/Saline	5	0	0	0	0	0
G2	DMH	13	124±34.3	61.7±9.6	33.1±9.0	18.4±9.2	10.8±6.5
G3	DMH/ASCex	14	27.2±6.7*	27.2±6.701*	0	0	0
G4	Control/ASCex	5	0	0	0	0	0

a, Means ± S.D.; *, Significant vs. G2 at P<0.05; AC, Aberrant crypt; n, No. of rats used for ACF counting

**Table 6:** Average Tomour Incidences (%), Multiplicities and Volumes

Group	Treatment	n	Tomour	Tomour Multiplicityb	Tomour Multiplicity	Tomour Volumes (cm^3^)
			Incidence (%)a	In tomour-bearing rat	In all ratsc	
G1	Control/Saline	5	0 0%	0	0	0
G2	DMH	16	15/16 (93.8%)^a^	31/15 (2.06±1.05)^b^	31/16 (1.94±1.05)^c^	0.34±.80
G3	DMH/ASCex	15	4/15 (26.7%)*	8/4 (2±1.41)*	8/15 (0.53±1.41)*	0.098±.091*
G4	Control/ASCex	5	0	0	0	0

a, Incidence: No. of tomour-bearing rats/ No. of rats per group (%); b, Multiplicity: Total No. of tomours / No. of tomour-bearing rats; (c), Percentage of rats bearing tomours in test group, tomour volume (v), the long (L) and short (w) dimensions (mm) of each tomour mass (length and width) were measured with calipers and calculated according to the equation: v= (L X W2 /2) (Shen et al. 2004); n, number of rats evaluated for tumour numbers.

**Table 7:** qRT-PCR Data Analysis of Rat *Cu/Zn* SOD Gene Relative to GAPDH Endogenous Gene.

Groups	Replicate Name	Cu/Zn SOD CT	GAPDH CT	ΔCT	ΔΔCT	Cu/Zn SOD Relative conc. (RQ =2^-ΔΔCT^)
G1	control	12.55	13.93	-1.38	0	1
G2	DMH	11.54	13.075	-1.535	-0.155	1.915
G3	DMH/ASCex	11.32	11.74	-0.42	0.96	0.625*
G4	ASCex	13.075	14.175	-1.1	0.28	0.83*

a, Each value is represented as means of 6 samples in duplicates; b, Values of RQ are expressed as means ± S.D. CT: Threshold cycle; ∆CT, Cycle threshold of* SOD1*–Cycle threshold of *GAPDH*; ∆∆CT, ∆CT - ∆CT (Control); RQ: Real-time quantitative PCR; *, Significant vs. G2 at P<0.05.

**Table 8:** qRT-PCR Data Analysis of Relative Concentrations of Rat *NF-κB* Gene Relative to *GAPDH* Endogenous Gene

Replicate Name	NF-κB CT	GAPDH. CT	ΔCT	ΔΔCT	NF-κB Relative conc. (RQ =2^-ΔΔCT^)
G1 control	22.41	13.075	9.335	0	1
G2 (DMH)	12.55	23.225	-10.67	-20.01	1671131.25
G3 (DMH+ASCex)	11.32	21.735	-10.41	-19.75	956036.955*
G4 (ASCex)	13.07	23.545	-10.47	-19.8	916359.675*

a, Each value is represented as means of 6 samples in duplicates; b, Values of RQ are expressed as means ± S.D. CT, Threshold cycle; ∆CT, Cycle threshold of *NF-Κb –*Cycle threshold of *GAPDH*; ∆∆CT, ∆CT - ∆CT (Control); RQ: Real-time quantitative PCR; *, Significant vs. G2 at P<0.05.

## Discussion

The present study has shown potent therapeutic effect of the aqueous extract from the ascidian Styela plicata (ASCex) against rat colon cancer initiated by 1,2-dimethyl hydrazine (DMH). The tumors’ incidences, multiplicities and sizes were significantly reduced after treatment with ASCex with no obvious side effects. In addition, treatment with ASCex has sharply inhibited the average total numbers of the aberrant crypt foci (ACF), colon cancer preneoplastic lesions, particularly large ones. ACF are well known estimators for colon carcinogenesis which are used as end point marker in cancer chemoprevention interventions and indicators for carcinogenesis. They steadily appear in the normally- appearing colorectal mucosa of colon cancer patients (Ghosh et al., 2018) or after DMH administration in rodents (Caetano, 2018). On the other hand, the inhibitory effects of the ASCex here are accompanied with a significant inhibition in the PCNA-LI (%) in colonic mucosa and in tumors. Martinez-Garcia et al., (2007) have shown that crude extract from the adult Ascidian, Cysodytes dellechiaji had significant antiproliferative activity against different tumor cell lines such as Colon H-116, breast SKBR3, pancreatic cancer PSN-1 and lung, A-549.

In the last few years, emerging evidence has been accumulating that marine organisms possess a high number of natural products with high medical importance (Li et al., 2019). The Mediterranean Ascidia, Styela plicata are protected by thick envelope, the tunic, composed of acidic mucopolysaccharides and different chemical and biological compounds. The tunic also comprises calcareous spicules which gather many cytotoxic materials such as pyridocridine alkaloids, diterpines, sphingosines and caramides (Di Bella et al., 2015). Palanisamy et al., (2017) recognized the differences in chemical constituents of methanolic extracts of Styela plicata and revealed the occurrence of 71 metabolites, amongst these alkaloids, fatty acids and lipids are the most dominant chemical groups, while most of these were difficult to detect precisely. They confirmed antitumor activities of the crude extract on Hela (cervical cancer), HT29 (colon cancer), MCF-7 (breast cancer) and M14 melanoma cancer cells. We have recently reported the most abundant active compounds in the aqueous and ethanolic extracts of Styela plicata obtained from the Egyptian Mediterranean coast (EL-Gamal et al., 2018). The same aqueous extract is used in the present study. Below we discuss the actual role of each chemical constituent and its possible inhibitory effects on colon cancer.

One of the most abundant compounds in the present ascidian aqueous extract is dermatan sulphate. Previously, the dermatan sulphates isolated from Phalusia migra and Styela plicata effectively attenuated matastasis of both MC38 colorectal adenocarcinoma and B16/BL-6 melanoma cells and the infiltration of inflammatory cells in a thioglycollate peritonitis mouse model (Kozlowski et al., 2011). As well, dermatan sulphate was suggested to play a pivotal role in the growth observed in SKBR3m breast cancer cell line, as the Golgi-specific labeling confirmed the localization of dermatan sulphate-epi1 in these cells at the Golgi apparatus, indicating that the location of the enzyme was a determinant for the synthesis of dermatan sulphate (Listik et al., 2020).

Another compound, Actinobolin, was found among the highest concentration of chemical compounds in the ethanolic ASCex detected by GC-MS chromatography (El-Gamal et al., 2018). Actinobolin is an antibiotic synthesized from the culture broths of Pseudomonas sp and produced by a Streptomyces where in mammalian cells it was shown to act directly by interfering with the binding site of the ribosomes with the amino-acyl tRNA in tumour cells (Chida and Sato, 2014). Actinobolin, was previously evaluated for antitumor activity against murine leukemias L1210 and P388 and was also shown to possess antitumor activity against i.p. implanted rat AH44 and AH7974F hepatomas (Okumoto et al., 1980).

Kuanoniamine A was also found in the present aqueous ASCex. Kuanoniamines A,B,C,D were first obtained from purple tunicates (Polycarpa aurata) and its mollusk pillager Chelynotus semperi (Anthony et al., 1990). Kuanoniamines were previously found to down regulate the proliferation of KB (human pharyngeal cancer) cell lines in vitro at an IC50 of 1 μg/ml (Utkina and Sagitolm, 2015). Also, Kuanoniamines A and C were found effective on the in vitro growth of five human tumour cell lines: MCF-7 (ER+), MDA-MB-231 (ER-), SF-268, NCI-H460, UACC-62, and on the non-tumour cell line MRC-5 after a continuous exposure of 48 h. The mechanism was suggested to be through its clear effect on DNA biosynthesis and MTT-reducing capacity as well as on a cell cycle (G2/M) progression and apoptosis (Kijjoa et al., 2007).

We also found abundant Lissoclinamide is in the present aqueous ASCex (El-Gamal et al., 2018). Lissoclinamide comprise a family of cyclic peptides first isolated from the ascidian Lissoclium patella, which showed previously significant anticancer as well as other pharmacological properties against human fibroblast and bladder carcinoma cell lines, as well as on normal lymphocytes (Negi et al., 2017).

Another important component, paromomycin is indicated as one the main compounds in the water ASCex. It is an antibiotic first isolated from the symbiotics living commensally with the marine tunicates and also isolated from Streptomyces krestomuceticus (Andrew, 2013). It is used nowadays an essential medicine against amebiasis, giardiasis, leishmaniasis, and tapeworm infection (Davidson et al., 2008). It has also been used for anti lieshmanial, anti-Cryptosporidium (Asadpour et al., 2018) and antitrichomonal (Henien et al., 2019). Paromomycin is a protein synthesis inhibitor in non-resistant cells by binding to 16S ribosomal RNA in prokaryotes (Cheuka et al., 2016) and in human and eukaryotic ribosome (Cheuka et al., 2016; Prokhorova et al., 2017). Thus the possible interaction of promamycin in the anticancer activity pathways of colon cancer here could possibly be through its inhibitory contribution to multiple aspects of the translation mechanism. This deserves further verification.

On the other hand, the aqueous ASCex exhibited relatively high levels of glycine sulphate. The significance of glycine sulphate in the ASCex is not well understood. However, it is well known that serine-glycine biosynthesis disturbs cellular growth and antioxidative ability, thus supportive to tumour homeostasis. The hyper-activation of the serine/glycine biosynthesis promotes tumorigenesis, while its deactivation inhibits cancer growth (Amelio et al., 2014). In addition, glycine is an essential constituent of glutathione, the principal antioxidant enzyme within the cell, which is required to keep the cellular redox balance. In the mitochondria, glycine also fuels heme biosynthesis and thus sustains oxidative phosphorylation (De Salvo et al., 2014). It was recently shown that both glycine consumption and expression of enzymes in the mitochondrial glycine biosynthetic pathway correlate with the rate of proliferation of cancer cells (Jain, 2012). Antagonizing glycine uptake and its mitochondrial biosynthesis preferentially impaired rapidly proliferating cells; in particular, silencing the mitochondrial SHMT2 gene and deprivation of extracellular glycine slowed down proliferation in HeLa cells and other fast-proliferating cancer cells by prolonging the G1 phase of the cell cycle (Jain, 2012). Thus, the involvement of glycine sulphate in colon cancer regression here could possibly be due to its antagonizing action against cellular glycine uptake and/or its involvement in the cellular antioxidant properties.

Other compounds found excessively in the present water extract of Styela plicata are palmitic and stearic acids. Palmitic acid is the most common saturated fatty acid found in animals, plants and microorganisms (Gunstone et al., 2007). Palmitic acid was previously shown to inhibit insulin pathway in hepatoma malignant cells (Zhang et al., (2012) depending on oxidation of the acyl-CoA which needs complete insulin receptor expression (Ruddock et al., 2008). The insulin growth factor receptor 1 (IGF1R) is involved in cell proliferation, differentiation, apoptosis and general anabolic cell practices and is markedly induced in CRC (Bowers et al., 2015; Walkiewicz et al., 2018).). Also, rats fed a diet of 19% palmitic acid and 56% carbohydrate for extended periods showed alterations in insulin secretion (Benoit et al., 2009). Taken together, the fact that palmitic acid found in the ASCex used hare has a significant role on regulating IGF-1 or its receptors and subsequently enhances the chemopreventive activity of ASCex against colon cancer is highly suggested.

Moreover, stearic acid was found in the water extract of the present ASCex. Stearic acid is one of the most abundant saturated fatty acids found in food after palmitic acid (Gunstone et al., 2007). Stearic acid was found correlated with lesser LDL cholesterol levels in human subjects (Hunter et al., 2009). A diet containing stearic acid with high resistant starch inhibited colon carcinogenesis in rats (Zhao et al., 2011). Recently, stearic acid nanoparticles conjugated with xylan have been developed for an effectual transfer of 5-fluorouracil (5-FU) in cancer remedy (Sauraj et al., (2019). Thus, stearic acid could have been effective in the present colon cancer therapeutic effects of ASCex in rats.

In conceivable confirmation for the premises of the interaction of the above mentioned survey for the natural mixture of chemicals found in ASCex on colon cancer therapy and its relation to the quantitative mRNA expression of NF-κB which is a gene responsible for DNA transcription and protein synthesis, and in correlation with a potent antioxidative stress marker gene, Cu-Zn SOD are evaluated. NF-κB is a protein complex that control DNA transcription, oncogenes, integrity and proliferation. When activated by reactive oxygen species (ROS), the NF-κB is highly expressed rendering the NF-κB to transfer to the nucleus to modulate target genes expression as NF-κB correlates with oxidative stress modulation (Perkins, 2007). The question whether ASCex could affect protein synthesis and modulate oxidative stress defense gene expression was answered in the present study.

Free radicals or reactive oxygen species causing oxidative stress have been evidently implicated in the incidence and progression of several health conditions such as cancer (Giustarini and Tsikas, 2009). Cu-Zn SOD gene the first line defense against ROS, an important anti-oxidant because it has a high catalytic efficiency, meaning it is fast produced to reduce the excessive reactive oxygen radical levels (Deepa et al., 2019). As expected, the normalized mRNA expression levels of NF-κB and Cu-Zn SOD genes were found significantly elevated after the stress caused by the carcinogen, DMH. Treatment with ASCex after DMH has sharply modulated this increase into almost normal levels. No side effects were noticed when animals were treated with the ASCex alone. These results are confirmatory to the present data of cellular proliferation data estimated with the PCNA-LI (%) in normally-appearing mucosa and in tumours.

It seems that the chemical constituents found in the ASCex has modulated the protein synthesis signaling factors and activated the synthesis of SOD, the first defense enzyme for antioxidative stress, and activated many other genes responsible for cellular differentiation and integrity in the colonic epithelium through NF-κB, thus reduced the tumour growth. In relation to the present findings, a number of studies indicated that deregulation of protein synthesis is a major contributor in cancer initiation (Błaszczak-Świątkiewicz, 2019) and metastatic progression (Graff and Zimmer, 2003). Presumably, a therapeutic key of ASCex here is achieved due to the higher requirement of transformed cells for protein synthesis in cancer, and translation regulation of some of the proteins involved in cancer progression, as well as inducibility for resistance against oxidative stress.
